# Community Management That Works: How to Build and Sustain a Thriving Online Health Community

**DOI:** 10.2196/jmir.2501

**Published:** 2013-06-11

**Authors:** Colleen Young

**Affiliations:** ^1^Canadian Virtual HospiceToronto, ONCanada

**Keywords:** online community, virtual community, health, patient, health care professional, community management, community strategy, guidelines, tutorial, peer-to-peer health

## Abstract

Health care professionals, patients, caregivers, family, friends, and other supporters are increasingly joining online health communities to share information and find support. But social Web (Web 2.0) technology alone does not create a successful online community. Building and sustaining a successful community requires an enabler and strategic community management. Community management is more than moderation. The developmental life cycle of a community has four stages: inception, establishment, maturity, and mitosis. Each stage presents distinct characteristics and management needs. This paper describes the community management strategies, resources, and expertise needed to build and maintain a thriving online health community; introduces some of the challenges; and provides a guide for health organizations considering this undertaking. The paper draws on insights from an ongoing study and observation of online communities as well as experience managing and consulting a variety of online health communities. Discussion includes effective community building practices relevant to each stage, such as outreach and relationship building, data collection, content creation, and other proven techniques that ensure the survival and steady growth of an online health community.

## Introduction

In her keynote address to participants of Medicine 2.0’11, Susannah Fox pointed out that “Patients and caregivers know things–about themselves, about each other, about treatments - and they want to share what they know to help other people” [1]. Thanks to the social Web, people facing a new diagnosis, undergoing treatment, or living with chronic illness can tap into larger networks. People are increasingly joining online health communities to share information and find support [2]. Fox went on to say, “Pew Internet research—and technology innovators—have found that if you can enable an environment in which people can share, they will and the benefits will entice others to join” [1].

Recognizing the potential benefits and increasing popularity of online patient support [2-7], more and more health care institutions and organizations are adding social Web elements to their repertoire of patient and family support and care provider collaboration tools. But social Web (Web 2.0) technology alone does not create a *successful* online community. A successful community is one in which members participate actively and develop lasting relationships [8]. Building and sustaining a successful community requires an enabler [9] and strategic community management [8,10].

This paper describes the community management strategies, resources, and expertise needed to build and maintain a thriving online health community; introduces some of the challenges; and provides a guide for health organizations considering this undertaking. The tutorial draws on insights from an ongoing study and observation of online communities as well as experience managing and consulting a variety of online health communities (see Table 1).

**Table 1:** Online communities built and used in this research.

Community	Description		Date of inception	Number of members^a^	Stage
SharingStrength/ FortesEnsemble [11]	A Canadian online resource library and community for women with breast cancer		Mar. 2007	1050	Mitosis (archived & adopted)
Health Care Social Media Canada [12]	A community of practice for people interested in exploring social innovation in health care		Sept. 2010	6564	Maturity
CancerConnection/ ParlonsCancer [13]	Canadian Cancer Society’s online community for people touched by cancer (H. Sinardo community manager)		Jan. 2011	2100	Establishment
Canadian Virtual Hospice/ Portail canadien en soins palliatifs [14]	Online resources and community for people living with limited time, losing someone, caring for someone, or working through grief		Dec. 2011	477	Late Inception

^a^Membership numbers as of Feb. 28, 2013.

At the time of writing this paper, the author (CY) manages the online communities of Health Care Social Media Canada (hcsmca) [12] and Canadian Virtual Hospice/Portail canadien en soins palliatifs [14]. Hcsmca was founded September 2010 and has since grown to become a mature community of practice of over 6500 members from all sectors of health care (health care professionals and professional associations, patients, caregivers, patient organizations, public health institutions, health educators, researchers, policy makers, communicators, and many more). Unlike the other communities described in this paper, hcsmca relies on social media platforms such as Twitter and LinkedIn for its online interactions rather than online community software.

**Figure 1:**
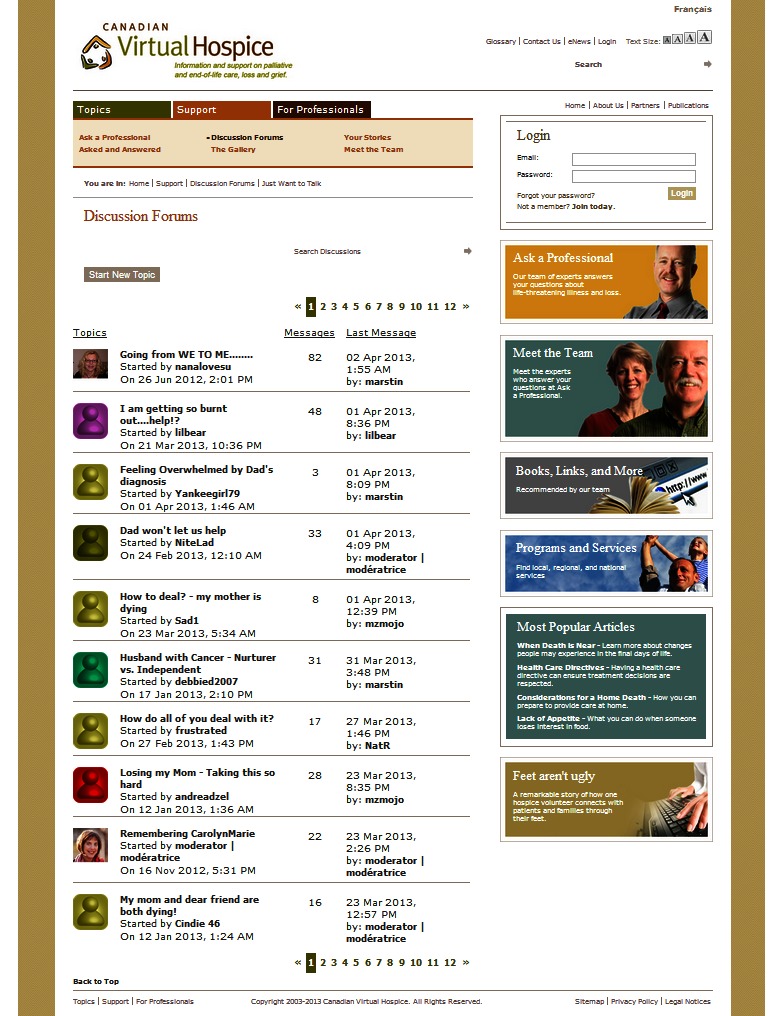
Virtual Hospice forum activity (captured April 2, 2013).

Virtual Hospice’s online community was first introduced in 2004, but after the initial launch, the discussion forums languished with little to no activity. In 2011, the author joined Virtual Hospice’s team to develop and implement a strategic online community management plan and establish a successful community. Today, the fledgling, but active, community continues to grow, providing peer-to-peer support and information for a very specific point in the health continuum, namely for people living with life-threatening disease, for friends and family who care about and for them, and for people dealing with grief and loss (see Figure 1).

SharingStrength/FortesEnsemble was a Canadian online resource library and community for women with breast cancer. The website was archived in 2011 [11] and the thriving English and French communities were successfully adopted by the Canadian Cancer Society’s then new online communities CancerConnection and ParlonsCancer [13] for people touched by cancer. During the adoption phase, the CancerConnection/ParlonsCancer communities were a welcoming new home and the presence of SharingStrength/FortesEnsemble’s moderator (the author) helped ease the transition of SharingStrength/FortesEnsemble members. Additionally, SharingStrength/FortesEnsemble’s community management practices and experience were transferred through collaborative moderator team training, the development of a moderation manual, and the creation of content for and with the community. Strategic development of CancerConnection/ParlonsCancer continues. Sustained collaboration between the community managers of CancerConnection/ParlonsCancer and Virtual Hospice helps members who are entering palliative care, or are caring for someone at the end of life, and who may wish to access the resources and services at Virtual Hospice or join that community.

Throughout the paper, the term “online community” is used; some researchers use “‘virtual community”. While the terms are interchangeable, online community is more widely used [15].

## What Is an Online Community?

As yet, there is no universally accepted definition of online community. Howard Rheingold, who coined the phrase virtual community, describes them as “cultural aggregations that emerge when enough people bump into each other often enough in cyberspace” [16]. A variety of disciplines have studied online communities, each one providing its own definition. Reviews by Preece and Maloney-Krichmar [17] and Iriberri and Leroy [8] illustrate how broadly defined online community is in this rapidly growing field. While Preece defines online community as “any virtual social space where people come together to get and give information or support, to learn, or to find company” [15], in this paper, an online community is a group of people who share a strong common interest, form relationships, and interact online. This definition encompasses three of the elements required for a successful online community discussed later in this paper:

- share strong common interest = domain
- form relationships = sense of community
- interact online = activity

There are different types of online communities [18]. People may form a community around a common interest, place, action, practice, or circumstance. While many of the strategies and tactics outlined in this discussion apply to all types of communities, specific examples focus on health communities. (Multimedia Appendix 1 lists all the online health communities mentioned in this paper.) Most health- and disease-based communities can be described as communities of circumstance. Rather than seeking online social interaction about a chosen interest, such as gardening (GardenWeb [19]) or citizenship (BritishExpats [20]), patients and caregivers are motivated to seek online interaction when a circumstance is imposed upon them, such as a new diagnosis or a change in health or well-being. They want to learn about their disease, get support, be less afraid of the unknown, and help others in similar circumstances [18]. Community members who have had similar experiences and can respond empathetically [21] may encourage strong relationships to develop, making patient and caregiver communities some of the most important on the Internet [18].

## Why Some Communities Succeed and Others Fail

Some online health communities thrive, sustaining activity for many years. Others languish, resembling ghost towns [22]. Their asynchronous tools, such as discussion forums (also known as message boards), often have multiple threads with few messages and last-post dates that are long past. Synchronous online chats and events are not well attended. Why do some communities succeed where others fail?

The success or failure of an online community depends, in part, on an organization’s commitment to sustained organizational and financial support for dedicated community management. Many health organizations are most concerned about the proliferation of misinformation [18]. While monitoring for misinformation through moderation and community watch mechanisms is important [3,23], developing and sustaining a successful online health community requires more than reactionary observation.

To build a thriving online community, organizations must ensure they have organizational commitment and the financial and human resources to not only start an online community, but also to support its growth and to evolve with the community throughout its life cycle. There are many flourishing online health communities. Before deciding whether to start an online community, resource-strapped health organizations should perform an environmental scan and consider whether their proposed community differs from those that exist or whether it makes more sense to seek collaborative opportunities with an established community. Investment in community management is imperative to an online community’s success [24]. Furthermore, an organization needs to

- establish and understand the domain of the proposed community [9]
- develop and sustain a community management strategy according to the community’s life cycle stage [8]
- foster a sense of community [25]

### The Domain

The *domain* is the common ground and sense of common identity upon which a community is built. Clearly defining the domain—whom the community is for, and why—affirms its purpose and value to both the community members and other stakeholders. The domain inspires people to participate in and contribute to the growth of the community [9]. In the case of health communities, domain may simply be a disease, such as cancer (CancerConnection [13]) or Crohn’s disease (Crohnology [26]), or life stage, such as birth (BabyCenter [27]) or death (VirtualHospice [14]).

An online community’s purpose and membership motivation also help determine which management strategies and platform tools will most likely contribute to its success [8,28]. In the case of online health communities, the purpose is usually to provide support and information to help people manage their health and well-being. Given that members are motivated by their need to receive emotional support, to reciprocate support, to learn, and to offer practical information, discussion forums are often the first platform tool introduced to a community platform. As the community evolves, the community manager may add tools that help members build a knowledge base or aggregate the community’s collective knowledge, such as a wiki to develop an e-book, group areas focused on a particular topic, or blogs led by specific community members.

### The Community Life Cycle

Reviewing online community research, Iriberri and Leroy recognized that online communities evolve through distinctive life cycle stages: inception, creation, growth, maturity, and, sometimes, death [8]. Iriberri and Leroy developed a framework of recommendations for success relevant to the developmental stage of the online community. This framework has been widely adapted and refined by community management practitioners to build successful online communities and will be discussed in more detail later in this paper.

### Sense of Community

Successful communities possess a strong sense of community, which comprises four elements [29]:

- Membership: Individuals have a feeling of belonging to, and identify with, the community.
- Integration and fulfilment of needs: The goals of individuals match those of the membership as a whole. As members satisfy their own needs, they also meet the community’s needs.
- Influence: Members feel they matter within the community and that they can influence and be influenced by the community.
- Attachment: Members share an emotional connection. Members believe they share or will share common history, places, or experiences.

Historically, measures of the sense of community in online communities have been adapted from McMillan and Chavis’s widely used measure of sense of community for face-to-face communities [30]. Evaluating these four elements in an online community offers insight into whether the online interaction can be defined as merely an online “settlement” [25], such as comments on a Facebook fan page, or a true online community, where members display actions reflecting feelings of belonging, influence, and attachment, and both their own as well as the community’s needs are being fulfilled. The Association of Cancer Online Resources (ACOR) [31] is a prime example of a true online community. ACOR is one of the oldest (established in 1995) and arguably the best known of online cancer communities, with a long history of sustained activity and a large body of reputable collective intelligence of hundreds of patients and caregivers.

While similarities exist between the sense of community of face-to-face and online communities, there are significant differences [30]. Given the increasing popularity of online communities and social networking, more research in this area would be useful. Blanchard’s Sense of Virtual Community measure represents a significant first step [30].

## Guidelines for Building and Sustaining a Thriving Online Community

### The Community Life Cycle

**Figure 2:**
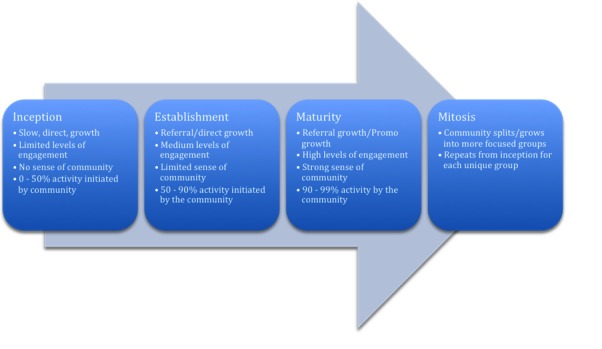
Life cycle of an online community (by Richard Millington).

Depending on a community’s developmental phase, its community management goals, strategies, priorities, and basic tasks will evolve. Figure 2 illustrates Richard Millington’s adapted version of the life cycle of an online community based on Iriberri and Leroy’s research [8,10]. This section introduces Millington’s four stages and explains how they can be used to build an online health community. It also expands upon the mitosis stage, examining the possibility of arranging for the adoption of a community and how to avoid the death of a community.

Understanding a community’s life cycle stage by monitoring its growth and activity (ie, posts, chats, events, and private messages) will help community managers decide when to adjust their community tactics and what changes the community would welcome and will enable them to substantiate the proposed evolutions for stakeholders. A community’s life cycle is not linear and managing its growth, activity, and design is an iterative process that must adapt to the needs of the members and the community’s purpose.

### Inception

The inception stage of an online community begins as soon as an organization begins to engage potential community members [32]. During the inception phase, the community manager

- develops relationships with potential members
- invites them to join and participate and encourages them to remain active
- helps establish tone and style
- recruits and nurtures an active core group to be community ambassadors

At the beginning of the inception stage, most of a community manager’s time is dedicated to making connections and building a core group of active members. This work should start even before the community platform is available. Community managers seek potential members by tapping into their personal and professional networks and their organization’s networks, monitoring discussions on social media, and recruiting volunteer participants. In other words, online community building starts with many one-on-one interactions, many of which occur offline. As Millington points out, “Getting your first 50 members is really hard work. It’s much harder than you probably imagine. In fact, earning those first 50 people is a full-time job” [32].

**Figure 3:**
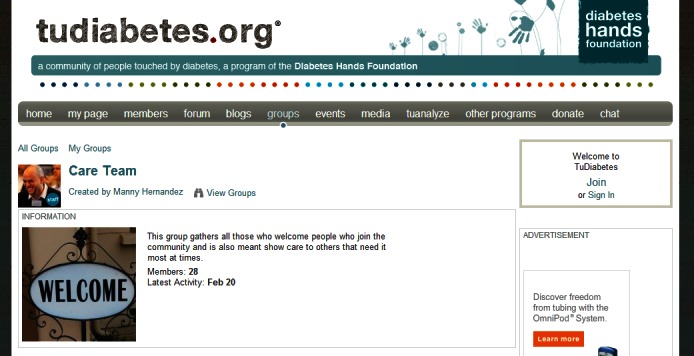
Screenshot from Tudiabetes (they created a group for volunteers, who ensure new members are welcomed).

Many health organizations have a volunteer corps. Volunteers can be recruited and offered training on how to use the community platform and how to foster supportive conversation online (see Figure 3 for an example of a volunteer group, the “Care Team” from Tudiabetes [33]). Volunteers can seed the community—engage in conversations among themselves—to ensure there is activity before new potential members are invited to the online community. It is vital to have activity in the community, especially in health communities, because no one wants to seek support from, or ask a vulnerable question into, a void [34].

Engaged volunteers welcome new members and ensure they receive timely—ideally immediate—replies. Reducing the time between posting a message and receiving a response encourages new people to stay engaged with the community members and to become committed members [35]. Receiving a prompt reply to a comment (within minutes) is far more memorable than receiving a reply hours or days later [36]. Implementing an opt-out notification system that alerts members by email when there is a new response to their contributions promotes quick response by both new and core members and can increase participation and activity [36].

Core members, including volunteers, play a vital role in the success of a community. They provide activity, but they also establish the tone of the conversation, welcome newcomers, connect people in the community, give lurkers (people who read but do not post) the confidence to join the conversation, and invite people to the community. Core members also help make improvements to the community, such as identifying possible barriers to participation and usability problems. In the inception stage, all new members are potential core members. Community managers have the opportunity to demonstrate to early adopters that they matter and that they have influence; managers should also ensure that there are channels for members to give their feedback. In the beginning, many members join because of a direct invitation. Beyond asking them to join, community managers should guide new members as to what they can do, for example, respond to a particular thread, start a blog on a given topic, add to the profile page, etc.

During the inception stage, slow, steady growth is best. Successful community managers integrate a few members at a time and work at converting visitors into active members. Thus the goal is not to accrue a large number of registrants, but rather to steadily grow the number of members who are committed to participating [32]. As activity and membership grow, the community manager will

- observe behaviors and make adjustments to usability, design, and strategy according to member feedback and behavior
- look for on- and offline event opportunities, such as live chats or in-person meetings
- increase activity and nurture a sense of community
- moderate posts and remove or correct misinformation

As membership and activity grow, community management strategies will gradually shift to establishment-stage activities.

### Establishment

The establishment phase of the community’s life cycle begins when members generate more than 50% of the activity and ends when they generate most (90%) of the growth and activity and when the sense of community starts to develop [32]. At this stage, the community manager focuses on modified versions of the inception-phase tasks, such as

- adding, nurturing, and supporting core members
- increasing activity with an eye to deepening the sense of community
- continuing with the growth strategy and broadening outreach
- expanding community tools

**Figure 4:**
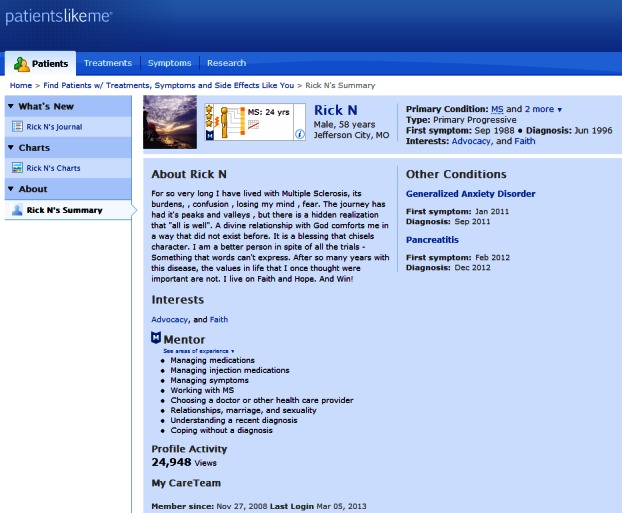
Profile of a member who has achieved mentor status on PatientsLikeMe site.

Activity remains the prime focus of the establishment phase. Supporting, observing, and investing time in the community’s core members helps achieve the remaining goals of the establishment period, namely growth and a deepening sense of community [30]. As the community matures, trust and lasting relationships begin to emerge and the strengths of core members reveal themselves [37]. Community managers should capitalize on the roles that core members start to assume, assigning them ownership of these roles and rewarding them for their activity, such as by featuring them in a newsletter article or blog post or by developing a system of recognition awarding status, for example, highlighting the most helpful members or most viewed posts. In the community PatientsLikeMe [38], members receive profile stars to indicate the amount of health information they’ve shared (see Figure 4). The stars show other members who has shared how much. Once a member receives the maximum three stars, they can apply for mentor status. Note that the PatientsLikeMe model assumes that the more members share, the more they can understand about their own experience and presumably the more they help others to learn. However, quantity does not trump quality. A single quality post from a regular member can also have a significant positive effect on a community. Community managers may wish to highlight the best comments of the week or month to recognize influential members.

As their commitment deepens, core members become more invested in the community’s success. The community manager can encourage further organic growth by developing a word-of-mouth referral plan, leveraging the commitment of these community ambassadors [32]. As Millington points out, it is first important to understand what motivates people to recommend a community (give a referral). They may be motivated by the potential to

- increase their status within the community
- increase their status outside the community
- help others in a similar situation
- help build the community

The first two motivations can be satisfied by recognizing key members and their contributions as mentioned above. For the latter two, the community manager can implement mechanisms that cater to the “pay it forward” sentiment that is prevalent among patients and caregivers and is a key motivator for health communities. Online community members often have offline interactions with other patients and caregivers, health care providers, and support organizations. These interactions can be fertile ground for promoting the community and its benefits and for extending invitations to join. Community managers should encourage and facilitate these opportunities.

While direct invitations to join the community continue, the community manager’s outreach efforts can shift to include broader awareness tactics at this stage, such as writing about the community (submitting articles to relevant newsletters and posting to social media channels and related blogs) and making presentations at relevant conferences and events.

Creating content for and about the community intensifies the sense of community. Content examples include producing a community newsletter or community section in the organization’s existing newsletter, writing newsletter articles or blog posts about individual core members or inviting them to make a newsletter or blog contribution, or producing a video featuring the community and its membership.

New members and new activity bring new ideas for discussions, for new roles and responsibilities, and for the development of new tools, features, and technologies. Thus, during the establishment phase, the community manager also continues to

- moderate posts
- monitor growth and activity
- make usability, design, and strategy improvements according to member feedback and user behavior data
- create event opportunities

Gradually, the community will evolve to the maturity phase.

### Maturity

An online community’s maturity stage begins when more than 90% of activity and growth are generated by members [32]. Many successful communities oscillate between the establishment and maturity phases as members retire from the community and community management strategies circle back to inception and establishment phase tactics to spawn new activity with new members. In the maturity stage, the size of the community reaches its critical mass, activity continues, and a sense of community is well established. While mature communities are often considered self-sustaining, the need for community management strategy and activity remains. The community manager’s attention turns to

- training core members to assume roles to maintain activity
- stimulating referrals and promoting the community to new members
- intensifying the sense of community
- assessing and optimizing processes
- developing collective value

**Figure 5:**
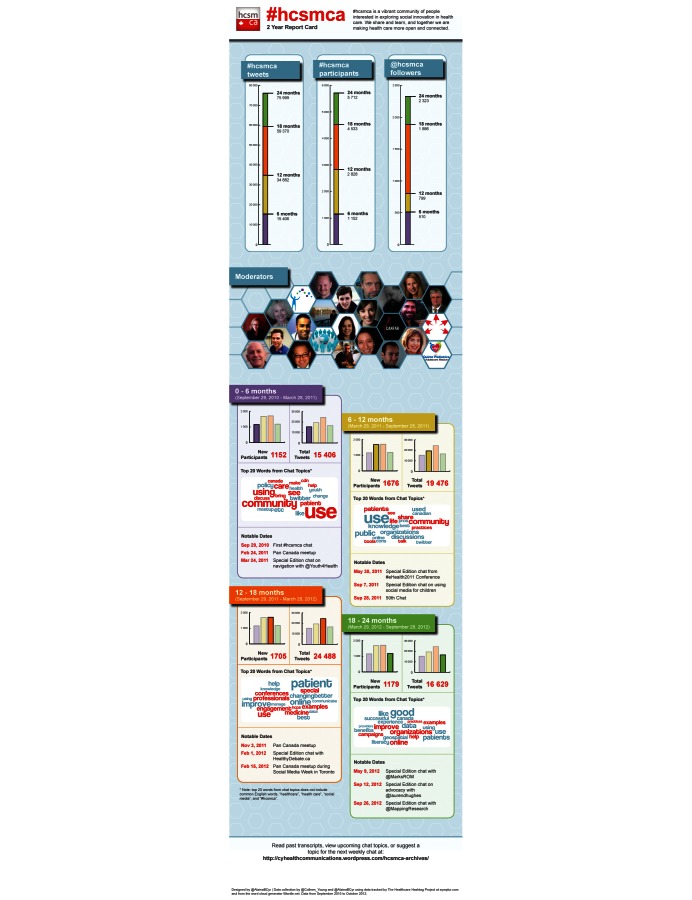
Infographic created by a hcsmca community member about the community.

The community manager should monitor the community and identify opportunities to develop collective value, which in turn intensifies the sense of community and will help promote the community. Collective value can be developed through co-creation, that is, involving community members to produce something together, such as a community charter or an e-book about the community or on a subject that represents the collective’s area of expertise. Members could be asked to write content for third-party publications or to respond to a survey to gather feedback on proposed improvements, giving them ownership of the changes. In the case of hcsmca, which is a mature community, members have been invited to become more involved, and some have taken initiative to create new roles and participation in co-creation of documentation and activities, for example:

- moderating weekly online chats
- organizing in-person meetings (meet-ups)
- writing blog posts about their experience in the community or about community activities
- analyzing the community (see Figure 5)
- creating documentation (eg, archiving of chat transcripts)

Many communities remain in the maturity stage for years and, if they maintain sustainable member size, may never reach mitosis or death [8,10].

### Mitosis, Adoption, Death

The mitosis phase begins when the community becomes largely self-sustaining and ends when activity and growth start to erode the sense of community [32]. This is a critical juncture in the community life cycle and community managers need to monitor activity and member attitudes and behaviors to ensure that the community does not become a victim of its own success. When a community grows to a point where the majority of members feel it is too large and too active, they will start to think they can no longer influence the community and they disengage. If the community manager does not do enough to mitigate this situation, the community will quickly die.

With proper monitoring and data collection during the establishment and maturity phases, however, the community manager will observe special interest groups emerging and will be able to create splinter groups or community subsets [10]. For example, a community for women with breast cancer may create a subcommunity of young women with breast cancer. While the word mitosis implies division into identical cells, a splinter community will develop its own culture and characteristics, not necessarily duplicating those of the parent community.

Each splinter community will return to the inception phase and progress through the life cycle. Because the community management efforts for each splinter community equal the effort of starting a new online community, initiating one splinter group at a time is advised [32].

Millington’s adapted version of Iriberri and Leroy’s online community cycle appears most applicable. It is practical and quite comprehensive; however, “Adoption” should be added to the life cycle for organizations that can no longer fund or otherwise support their thriving online community. Sometimes burgeoning or thriving online communities are forced to die a premature death when funding priorities shift or dry up despite the success of the community. In such cases, planned adoption can support the survival of the community of people. SharingStrength/FortesEnsemble is one example of successful adoption of a community when the funder’s priorities were redirected [11].

Like Iriberri and Leroy, Millington refers to the possibility of the final life stage: death. Understandably, however, his practice guidelines do not focus on this stage because he contends that good community management practices render this stage avoidable [39]. He writes, “You have a short window to reverse a decline before it becomes a death spiral”. Community managers can avoid the death spiral by monitoring metrics of growth, activity, and sense of community, in other words, measuring for success.

## Measure for Success

Metrics for quantifying the success of online communities vary widely [15,40]. In her paper from 2001, Preece suggests tracking determinants that measure community members’ sociability and the usability of the technology to measure success. Sociability refers to how members of a community interact with each other on the community platform or technology; usability is primarily concerned with how users interact with the technology. Analysis of a community at key points in its life cycle using rigorous determinants is highly recommended. For example, Preece outlines the evaluation of the purpose, people, and policy of a community (sociability) and information design, navigation, access, the dialogue and social supports like prompts, use of avatars, etc (usability) [15]. However, collecting such thorough data regularly is likely unrealistic for most community managers.

One can also apply many useful health-related metrics, such as self-efficacy and quality of life, to analyze online communities. For the purpose of this paper, the focus is on metrics specifically pertinent to the community manager’s role in developing a thriving online community. This role includes ascertaining the community’s needs and identifying relevant community trends and developments to steward the health of the online community—its growth, activity, and sense of community—and to foresee and fix negative trends before they become problems. Thus, it is important to monitor the trends and to gather data that will

- keep track of the growth of the community
- demonstrate activity and engagement (sociability)
- improve the community, discover problems, and validate what works (sociability and usability)
- report progress and demonstrate the value of the community to stakeholders, including community members

Community managers should collect a manageable amount of data regularly, consistently, and accurately over the life of their community. Millington recommends tracking active members to determine growth and engagement [41]. Growth data should include not only the number of registrants (members), but also the number of registrants who contribute (active members) and the number who made a contribution in the past month. Having a high number of registrants relative to the number of participants signals a low conversation rate and may indicate the existence of barriers to participation (usability). Knowing who has not contributed in the past month can inform targeted outreach activities.

Activity and engagement data help determine where the community is in its life cycle (a more engaged community equals a more developed community) and identifies potential problems early, when they can be more easily corrected. Activity and engagement can be assessed by monthly tracking of the number of posts, the average number of contributions per active member, the average number of responses to a post, the average time for a post to receive a response, and the average number of visits per active member [10]. By identifying the activity and engagement level, the community manager can validate the life cycle stage and introduce strategies and tactics appropriate to that stage. Managers can use the data to recognize the type of conversations and activities that generate the highest engagement, which member behaviors are most apt to lead to increased activity, and whether too few members are dominating the conversation.

Table 2 lists examples of growth and activity data that community managers could track monthly to monitor the health of their community. The data can be used to track trends and progress, to help identify which community management strategies and activities are working, and to improve those that are not. These data can also be used to develop tactical activities to promote growth and activity tailored to the community’s members, potential members, and technology.

**Table 2:** Examples of growth and activity data that community managers could collect.

Measurement (monthly)	Key questions
Number of first-time visitors^a^ to community	Are people finding the community? What outreach tactics can be used, or technology optimizations made, to increase the number of visits?
Number of new members (registrants^b^)	What is the conversion rate from visitor to member? Is there a usability barrier to registration? Is the platform optimized to motivate visitors to become members? Are outreach tactics attracting the kind of people suited to the community?
Number (or percentage) of active members^c^ who made a contribution (post)	What is the conversion rate from registrant to active member? What motivates people to participate (high conversion rate)? Why are people motivated to register, but not to participate (low conversion rate)?
Number of new active members	What influences are successfully motivating new members to participate and then to become active members?
Number of returning active members	Are an increasing number of regular members remaining active? Why or why not?
Total number of active members	Have new members become active members? Why or why not?
Total new posts	Is activity consistently increasing? Where is activity greatest (eg, discussion forums, blogs, groups, polls)?
Average number of contributions per active member	Is this number increasing or decreasing? Should more effort be dedicated to activity of existing members rather than growth? What activities are contributing to increased activity or not?

^a^Visitor = someone who has visited the community but has neither registered nor contributed.

^b^Member (registrant) = someone who has registered with the community but has not yet made a contribution.

^c^Active member = someone who has made a contribution within a determined period of time (eg, past month).

Periodic sample measurements of the community can also be useful. For example, calculating the number of contributions per active member over a given period can demonstrate whether the activity is shared among many or few members, identify the community leaders, and discover who is contributing infrequently. Speed of response, how much response, and what kind of response should be monitored. As mentioned earlier, the quicker that members receive a response, the higher the sociability of the community, which usually leads to more activity [35].

It can be useful to know who is participating in the community. However, gathering all demographic information during the registration process is ill advised—doing so can be a barrier to participation. Instead, members who fill out their profile can be used as a measurement of engagement and sense of community, ie, a measurement of initial successful engagement. Members who have not completed their profile provide an opportunity for community managers or recruited volunteers to contact them and obtain valuable feedback about why they have not completed their profile. Perhaps there is a usability issue or the member needs encouragement or mentoring to feel part of the community.

One could also consider surveying the community to assess its sense of community. As mentioned, Chavis et al developed a sense-of-community index, which Blanchard adapted for online community use in 2007 [30]. But as Millington points out, there is a limit to how often members will agree to be surveyed. Furthermore, members who voluntarily respond to surveys are often the most engaged community members and those who feel the strongest sense of community [10]. Such surveys will likely produce biased results. Nonetheless, community managers should find ways to gather feedback from members and ensure members have clear and simple channels to provide it.

## Challenges

Many health organizations are most concerned about disclosure of personal health or other sensitive information and the proliferation of misinformation [3,18,23]. However, experience and observation show that clear policies, proactive community management, and active moderation and community participation render these concerns largely unfounded for online communities associated with reputable organizations. It should be noted that some communities exist for the sole purpose of encouraging unhealthy behaviors, such as pro-anorexia groups [42]. This discussion refers to online communities that support healthy behaviors and how they manage transgressions of their terms of use.

Clearly stated policies make it easy for moderators to modify—and in some cases remove—posts that contravene terms of use, such as commercial postings, advertisements, or impersonations; posts that relate to illegal activity; those that contain disrespectful language, etc [43,44]. For example, Macmillan Cancer Support’s online community had to deal with a member posing as a cancer patient. In a blog post [45], the moderation team described the transgression to the community, expressed empathy for the upset it may have caused, explained how members can protect themselves, and gave an opportunity for the community to discuss the situation.

Community managers, moderators, and core members model behavior and can guide members who may have unwittingly shared sensitive information or misinformation. This modeling establishes and maintains the desired tone of a community. Communities with a secure sense of community can rely on responsive self-policing to correct misguided behavior and misinformation. In fact, rather than removing misguided information, allowing and enabling community members to correct misconceptions and provide balanced debate can be a very productive bonding opportunity that deepens the sense of community and establishes the value of collective knowledge. On hcsmca’s LinkedIn Group page, a marketing manager made a commercial post about her company’s upcoming patient experience conference that did not include patients. This contravened the community’s principle of including patients. The ensuing conversation demonstrated hcsmca’s community cohesiveness, resulted in an informative discussion, and deepened the community’s sense of purpose and influence [46]. Because the hcsmca community relies on Twitter for the majority of its online interactions, one might think it would be more susceptible to abuse with little recourse to correct misbehavior. However, the tight-knit nature of the community and its unified understanding of the community’s purpose guards its principles and guides the behavior of newcomers, quickly correcting or rejecting misuse.

Clinical study recruitment may be desirable in some health communities. If so, guidelines and criteria about how, what, and where to post for recruitment should be readily available for researchers to consult. Recruitment policies may change as a community matures. For example, a request for photo subjects was posted on Virtual Hospice when the community was just starting out [47]. The post was removed and the poster, who had no interest in becoming a community member, was invited to submit the request through more appropriate channels of the organization. In a more mature community, such a request may not have been inappropriate.

As these examples demonstrate, undesirable behavior does happen in online communities, but responsive community management can maintain the integrity, reliability, and value of the collective community knowledge.

The bigger issue challenging the success of online communities is the failure to recognize the time and effort required to build a thriving and reputable online health community. Building a community takes organizational commitment as well as sustainable financial and human resources throughout the community’s life cycle [48]. An evidence-informed community management strategy [49] and a dedicated, experienced community manager can ensure an online community’s success.

## Conclusion

Most of the practices discussed in this paper are not unique to health communities. However, establishing an online health community’s purpose and its members’ motivations helps community managers modify these practices to tailor the engagement tactics for online health communities. As people increasingly turn to online health communities for information and support, it is vital to realize that community management is more than just moderation (see Multimedia Appendix 2). Behind each thriving online community is an enabler—a community manager who establishes the tone, proactively initiates and maintains growth through outreach and by encouraging member referrals, ensures regular activity, nurtures core members, hosts events, creates content for and about the community, fosters a sense of community, and constantly gathers data and feedback to guide and improve the community. A successful online community manager will adapt the management strategy as the community evolves through the various stages of its life cycle. “Fostering participation is one of the most difficult, yet crucial, roles for online facilitators” [50].

## Multimedia Appendix 1

Online communities mentioned in this paper.

## Multimedia Appendix 2

A 12-point summary to building a successful online community.

## Abbreviations

ACOR: Association of Cancer Online Resources
hcsmca: Health Care Social Media Canada
