# Supplementary material for: Community Management That Works: How to Build and Sustain a Thriving Online Health Community
Source: J Med Internet Res. 2013 Jun 11;15(6):e119. doi: 10.2196/jmir.2501 (PMC3713910; doi:10.2196/jmir.2501)
Supplement: Supplementary file 1 [file jmir_v15i6e119_app1.pdf]

Multimedia Appendix 1. Online communities mentioned in this paper.

Community	Description	Supporting Organization	Community Manager
Health Care Social Media Canada [12]	A community of practice for people interested in exploring social innovation in health care	Volunteer led	Colleen Young
Canadian Virtual Hospice/ Portail canadien en soins palliatifs [14]	Online resources and community for people living with limited time, losing someone, caring for someone, or working through grief	Canadian Virtual Hospice	Colleen Young
SharingStrength/ FortesEnsemble [11]	A Canadian online resource library and community for women with breast cancer	SharingStrength	Colleen Young
CancerConnection/ ParlonsCancer [13]	An online community for people touched by cancer	Canadian Cancer Society	Heather Sinardo
Crohnology [26]	Social health network for people with Crohn’s and colitis	Healthy Labs, Inc.	Sean Ahrens
BabyCenter [27]	Online resources and community for new, expecting, and “actively trying” parents	BabyCenter Canada	N/A
ACOR [31]	Collection of 140+ online cancer communities	Association of Cancer Online Resources, Inc.	Gilles Frydman
Tudiabetes [33]	An online community of people touched by diabetes	Diabetes Hands Foundation	Manny Hernandez
PatientsLikeMe [38]	An online community of people sharing health experiences to help themselves, other patients, and organizations	PatientsLikeMe	Liz Morgan
MyMacmillan Online Community [45]	An online community for anyone affected by cancer in any way	Macmillan Cancer Support	Laura Goss
