# Supplementary material for: Community Management That Works: How to Build and Sustain a Thriving Online Health Community
Source: J Med Internet Res. 2013 Jun 11;15(6):e119. doi: 10.2196/jmir.2501 (PMC3713910; doi:10.2196/jmir.2501)
Supplement: Supplementary file 2 [file jmir_v15i6e119_app2.pdf]

Multimedia Appendix 2. 12-point summary to building a successful online community.

1. **Appoint a dedicated community manager.** Ensure your online community plan is supported by organizational commitment and sustained financial resources.
2. **Define the community domain.** Don't create a domain. Find a strong common interest that a lot of people care about and build a community around it.
3. **Develop a community management strategy** and make adjustments as the community evolves through its life cycle.
4. **Recruit and connect members** before you launch the online community platform.
5. **Start small.** Slow, steady growth is best. Integrate a few members at a time and work at converting visitors into active, committed members.
6. **Build strong relationships with committed (core) members.** Assign specific responsibilities to members of this core group according to their strengths. Foster ownership and a sense of community.
7. **Concentrate on activity** and sustaining momentum**.** A small, but active community will attract more activity and make it easier to grow the community.
8. **Model behavior.** Your posts and those of the core group set the tone and guide members to establish and adopt the community's etiquette.
9. **Create content for and about the community.** Write about the people in your community. Recognize individual contributions and collective achievements.
10. **Encourage member referrals.** Develop a referral plan. Let members recruit new members and promote the community.
11. **Allow and enable debate.** They make great conversations. Allow members to disagree and the community to self-correct. Promote the value of collective knowledge.
12. **Measure for success.** Collect data regularly, consistently, and accurately to improve the community, discover problems, and validate what works. Set up clear feedback channels.
